# MS/MS analysis of four scorpion venoms from Colombia: a descriptive approach

**DOI:** 10.1590/1678-9199-JVATITD-2020-0173

**Published:** 2021-07-09

**Authors:** Sebastian Estrada-Gómez, Leidy Johana Vargas-Muñoz, Monica Maria Saldarriaga-Córdoba, Arie van der Meijden

**Affiliations:** 1 Toxinology Research Group - Serpentarium, University of Antioquia (UdeA), Medellín, Antioquia, Colombia.; 2 School of Pharmaceutical and Food Sciences, University of Antioquia (UdeA), Medellín, Antioquia, Colombia.; 3 School of Medicine, Cooperative University of Colombia, Medellín, Antioquia, Colombia.; 4 Center for Research in Natural Resources and Sustainability, Bernardo O’Higgins University, Santiago, Chile.; 5 Research Center in Biodiversity and Genetic Resources (CIBIO), University of Porto, Vila do Conde, Portugal.

**Keywords:** Scorpion, Venom, Colombia, MS analysis, Toxins, Sodium channels

## Abstract

**Background:**

Scorpions are widely known for the neurotoxic effects of their venoms, which contain peptides affecting ionic channels. Although Colombia is recognized for its scorpion diversity, only a few studies are available describing the venom content.

**Methods:**

In this descriptive study, we analyzed the MS/MS sequence, electrophoretic and chromatographic profile linked to a bioinformatics analysis of the scorpions *Chactas reticulatus* (Chactidae), *Opisthacanthus elatus* (Hormuridae), *Centruroides edwardsii* (Buthidae) and *Tityus asthenes* (Buthidae) from Colombia.

**Results:**

Each scorpion showed a specific electrophoretic and chromatographic profile. The electrophoretic profiles indicate the presence of high molecular mass compounds in all venoms, with a predominance of low molecular mass compounds in the Buthidae species. Chromatographic profiles showed a similar pattern as the electrophoretic profiles. From the MS/MS analysis of the chromatographic collected fractions, we obtained internal peptide sequences corresponding to proteins reported in scorpions from the respective family of the analyzed samples. Some of these proteins correspond to neurotoxins affecting ionic channels, antimicrobial peptides and metalloproteinase-like fragments. In the venom of *Tityus asthenes,* the MS^n^ analysis allowed the detection of two toxins affecting sodium channels covering 50% and 84% of the sequence respectively, showing 100% sequence similarity. Two sequences from *Tityus asthenes* showed sequence similarity with a phospholipase from *Opisthacanthus cayaporum* indicating the presence of this type of toxin in this species for the first time. One sequence matching a hypothetical secreted protein from *Hottentotta judaicus* was found in three of the studied venoms. We found that this protein is common in the Buthidae family whereas it has been reported in other families - such as Scorpionidae - and may be part of the evolutionary puzzle of venoms in these arachnids.

**Conclusion:**

Buthidae venoms from Colombia can be considered an important source of peptides similar to toxins affecting ionic channels. An interesting predicted antimicrobial peptide was detected in three of the analyzed venoms.

## Background

Scorpion venoms have evolved over 400 million years into a complex, but well elaborated library of toxins that can differ dramatically in its effects among species [1]. The diversity of protein compounds (peptides, proteins and enzymes) and non-protein compounds (salts, neurotransmitters, etc.) make these venoms a promising source of molecules with antibacterial, antifungal, antiviral, antimalarial and anticancer activities [2-5], and a potential source for the design of new drugs [6,7]. The most active molecules displaying such activities are peptides that can be split into non-disulﬁde bridge (NDBP) and disulﬁde bridge (DBP) peptides, showing alpha helical linear motifs or inhibitory cysteine knots respectively. The NDBP compounds were reported recently and the main characteristic of these molecules is the lack of disulfide bridges, the cationic net charge, the sequence diversity, the hemolytic and antibacterial activity, and the relatively low molecular mass (1-4 kDa) [8]. Most of these peptides possess an amphipathic alpha-helical structure like those reported for different cationic antimicrobial molecules [3,8-15]. DBP are the major molecules described in these venoms and are characterized by containing around 30 to 70 amino acids residues and three or four disulfide bridges [3,8-15]. The major targets of these toxins are ionic channels like sodium (Nav), potassium (Kv), chlorine (Clv) or calcium (Cav) channels in the nervous system, blocking or gating the channel mechanism and thereby exhibiting a neurotoxic activity. 

Despite the diversity of scorpions in Colombia, only a few studies are available describing the venom content [16-19]. No studies have been found describing the venom content of *Chactas reticulatus, Opisthacanthus elatus, Centruroides edwardsii* or *Tityus asthenes*. The only available studies report the phospholipase A_2_ content and activity in the venom of *O. elatus*, and the intraspecific biochemical differences detected in the venom of *C. edwardsii* from two regions in Colombia [16,17]. The genus *Tityus* is probably one of the must studied scorpions in South America, but from Colombia the only available studies of this genus are the proteomic analysis of *Tityus pachyurus* reporting specific toxins affecting Na^+^ and K^+^ channels [18,20], and the peptide content description of *Tityus macrochirus* [19]. There is no study available on *T. asthenes*. Venom from *Ch. reticulatus* is still completely unexplored and the venom of this species had hitherto not been described or analyzed.

Here we report the first partial amino acid sequences including the post-translational modifications (PTM) of the venom from *Ch. reticulatus*, *O. elatus, C. edwardsii* and *T. asthenes*, with the respective electrophoretic and chromatographic profile with analysis of their predicted antimicrobial activity and the report of different partial toxins that may affect ionic channels.

## Methods

### Species selection

Scorpions with epidemiologic and clinical importance in Antioquia (North-west Colombian Andean region) according to Otero et al. **[** 21-23 **]** *Chactas reticulatus, Opisthacanthus elatus, Centruroides edwardsii* and *Tityus asthenes* (with no or scarce previous reports), were selected for this research and kept in captivity in the serpentarium of the University of Antioquia, Medellin (COLBIOFAR-149) with water *ad libitum* and fed with insects (*Periplaneta americana* and *Tenebrio molitor*). One specimen of *Chactas reticulatus* was sourced from the municipality of El Retiro (El Salado sector) at 2100 meters above sea level (m.a.s.l), while seven specimens of *Opisthacanthus elatus* born in captivity from an individual from the municipality of Remedios - Antioquia were used. Four specimens of *Centruroides edwardsii* were from two localities in the municipality of Amaga and Medellín at 1250 m.a.s.l. and 1450 m.a.s.l. respectively. Furthermore, six specimens of *Tityus asthenes* originating from the municipality of Carepa (Urban area) at 26 m.a.s.l. were used.

### Venom extraction

Venom extraction was carried out using electro-stimulation. Metal electrodes, wetted with a saline solution, were carefully positioned on the metasoma and a block signal with an amplitude of 18V at 40-60Hz was applied twice with an interval of 5 sec using a custom-made electro-stimulator (model 01). Collected venom was transferred to dry low-protein binding vials, freeze-dried and stored at -20°C until use. These procedures were in accordance with the ethical principles in animal research adopted by the World Health Organization for the characterization of venoms. After each extraction, all animals were kept alive in captivity. 

### Electrophoretic profiles

All electrophoretic profiles of crude venoms were analyzed using 12% sodium dodecyl sulfate polyacrylamide gels (SDS-PAGE) according to Laemmli [24], and stained with Coomassie blue R-250. Molecular weights were estimated using standard low range markers standards (Bio-Rad). Venoms were loaded at a concentration of 1.5 mg/ml and a final volume of 20 µL. Venom concentrations were assessed following the Biuret method using Bio-Rad Protein Assay reagent and bovine serum albumin (BSA) as standard [25-27]. A 3D scatterplot representation with the number of compounds detected in each venom based in their absence-presence in every species was performed using the software SIGMAPLOT v. 14 (Systat Software, San Jose, CA). To do so, compounds detected in each venom were grouped in four different ranges: 14 kDa to 31 kDa, 31 kDa to 45KDa, 45 kDa to 97.4 kDa, and above 97.4KDa, and plotted. Additionally, venoms showing potentially toxins affecting ion channels in the MS/MS analysis were run on 10% TRIS-TRICINE gels, and stained with Coomassie blue R-250. Molecular weights were estimated using standard broad range standards (Bio-Rad). Quantification of volumes and calculation of molecular weights were performed using the software GelAnalyzer 19.1, available at: http://www.gelanalyzer.com/ [28]. Molecular weights were calculated using the known values of the standard broad rank markers (Bio-Rad): 200 kDa, 116 kDa, 97 kDa, 66 kDa, 45 kDa, 31 kDa, 21 kDa, 14 kDa, 6 kDa. To estimate the molecular weight, we used a simple exponential fit approximation and according to the Rf (retention factor, measured as the band distance migrated/gel length) of each analyzed band. 

### Chromatographic profile

We followed the methodology proposed by Fernandez et al. [29] and adapted by Estrada et al. [16,17] for arachnid venoms separation. One milligram of whole venom was dissolved in 200 μL of solution A (0.1% trifluoroacetic acid - TFA, in water) and centrifuged at 3500 g. The supernatant was then applied to a reverse-phase RESTEK C18 column (250 × 4.6 mm), and separated on a Shimadzu Prominence HPLC. Proteins were eluted by a gradient towards solution B (0.1% TFA in acetonitrile - ACN 99%) as follows: 5% B for 5 min, 5-15% B over 10 min, 15-45% B over 60 min, and 45-70% B over 12 min at a flow rate of 1.0 ml/min. The chromatographic run was monitored at 215 nm and fractions were collected, freeze-dried and stored at -20 °C until used.

### Peptide mass determination by high-resolution LC-MS

For *Ch. reticulatus*, *C. edwardsii* and *T. asthenes* we selected the peaks with the best intensity and resolution from the RP-HPLC chromatograms. For *O. elatus*, we collected the major peak of the phospholipase region according to Estrada et al. [17], looking for a deeper characterization of this region. We wanted to see if this region exclusively contained phospholipase proteins or if there were more components co-eluting in the region. Selected dried fractions were digested and submitted to the MS/MS equipment as explained below.

### Sample digestion

Sequence grade Lys-C/Trypsin (Promega) was used to enzymatically digest the venom samples. The samples were reduced and alkylated. All digestions were carried out in the Barocycler NEP2320 (PBI) at 50°C under 20 kpsi for 2 hours. Digested samples were cleaned over C18 spin columns (Nest Group) and dried. Resulting pellets were re-suspended in 97% purified H_2_O/3% ACN/0.1% formic acid (FA). A volume of 5 µL was used for nano LC-MS/MS analysis.

### LC-MS/MS

Fractions were run on a nano Eksigent 425 HPLC system coupled to the Triple TOF 5600 plus (Sciex, Framingham, MA). The method used for analysis was 120 minutes at 300 nL/minute over the cHiPLC nanoflex system. The trap column was a Nano cHiPLC 200 µm x0.5 mm ChromXP C18-CL 3 µm x 120 Å followed by the analytical column, the Nano cHiPLC 75 µm x 15 cm ChromXP C18-CL 5 µm x 120 Å. The sample was injected into the Triple TOF 5600 and through the Nanospray III source equipped with an emission tip (New Objective, Woburn, MA, USA). Peptides from the digestion were eluted from the columns using a mobile phase A of purified H_2_O/0.1% formic acid (FA) and a mobile phase B of ACN/0.1 % FA. With a flow rate of 0.3 µL/min, the method started at 95% A for 1 minute followed by a gradient of 5% B to 35% B in 90 minutes and from 35% B to 80% B in 2 minutes. Eighty percent of B was held for 5 minutes before being brought to 5% B and held for 20 minutes. PTM are reported for each containing peptide.

### Data analysis

The data acquisition was performed monitoring 50 precursor ions at 250 ms/scan. Mascot Daemon v.2.4.0 (Matrix Science) was used for similarities searches against the different databases downloaded from the UniProt and NCBI websites. Data analysis was run in the Bindley Bioscience Center at Purdue University. Multiple sequence alignment was completed using the Clustal Omega software (http://www.ebi.ac.uk/Tools/msa/clustalo/) of MS/MS sequences from each venom with the respective similar peptide/protein.

### Bioinformatics analysis

The search for similar peptides/proteins matching KISSV[X]NKDKI peptide was performed in Protein Information Resource (PIR) databases [30,31]. Specifically peptide matching using Apache Lucene-based search engine [32], using as query sequence the peptide without specifying the residue in the bracket [X], and each of the following residues A,I,V,S and N according to MS/MS analysis. The search was performed in the databases UniProtKB/Swiss-Prot with isoforms.

### Evaluation of the physicochemical properties

The corresponding physicochemical properties of identified peptides following an *in silico* analysis, resulting in metrics for peptide length (residues), molecular weight, total hydrophobic ratio, net charge at physiological pH, and the Boman Index, were determined using the Antimicrobial Peptide Database Calculator and Predictor (APD3 http://aps.unmc.edu/AP/) [33]. 

## Results

### Electrophoretic and chromatographic profiles

**Figure 1.**
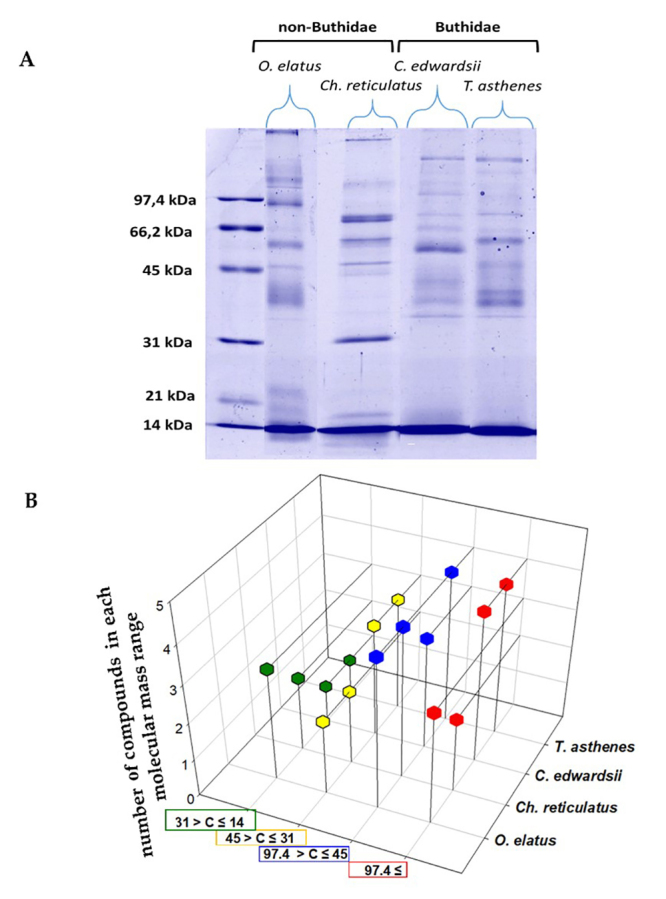
(A) Crude venom SDS-PAGE profile in a 12% gel under reducing conditions of *Opisthacanthus elatus*, *Chactas reticulatus*, *Centruroides edwardsii* and *Tityus asthenes*. (B) 3D scatterplot of the number of compounds found in each molecular mass range of every species. Both figures show visible differences among species in relation to electrophoretic pattern and number of compounds, with the *T. asthenes* and *C. edwardsii* profiles being more similar. Green dots indicate compounds with a molecular range between 14 kDa and 31 kDa. Yellow dots indicate compounds with a molecular range between 31 kDa and 45 kDa. Blue dots indicate compounds with a molecular range between 45 kDa and 97.4 kDa. Red dots indicate compounds with a molecular range of 97.4 kDa or above. Letter C: compound.

 The venom from each species showed a specific electrophoretic profile, and some differences were detected among the species (Figure 1A). Visibly, differences are specially observed in the high molecular mass compound (HMMC) region above 31 kDa (where HMMC like metalloproteinases are commonly found), with some compounds migrating close to 14 kDa and 21 kDa (where HMMC like phospholipases are commonly found) (Figure 1A). Venoms from C. edwardsii and T. asthenes (Buthidae) showed a very similar profile with the majority of compounds distributed above 31 kDa and with few compounds around 14 kDa (according to Figure 1B). The non-buthidae venoms from O. elatus, Ch. reticulatus shows electrophoretic profiles with visible differences; most compounds are distributed among 97 kDa and 45 kDa (see Figure 1B). A specific difference was observed below 31 kDa in the O. elatus venom, were at least 3 compounds were detected migrating close to 14 kDa (see Figure 1B). 

**Figure 2.**
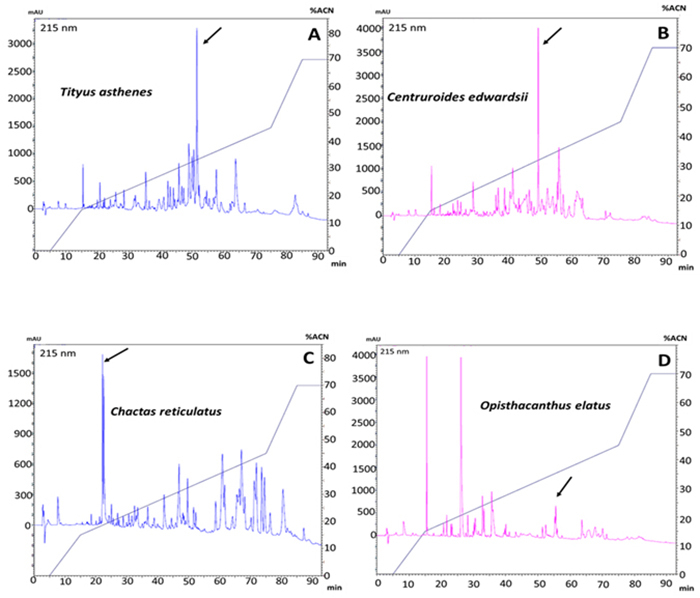
Venom chromatographic profile. RP-HPLC chromatographic proﬁles of the crude venom of all scorpions using a C18 column (250 mm-4.6 mm): **(A)** *T. asthenes*, **(B)** *C. edwardsii*, **(C)** *Ch. reticulatus* and **(D)** *O. elatus*. Elution gradient used: 0-70% of acetonitrile (99% ACN in TFA 0.1%). The run was monitored at 215 nm. Arrows indicate fraction subjected to MS/MS analysis.

As observed in the electrophoretic pattern, the chromatographic profile showed clear differences between venoms from each species (Figure 2). In all cases, we obtained complex chromatograms with good resolution and well defined peaks. As seen in the electrophoretic profile, venoms from C. edwardsii and T. asthenes (Buthidae) showed a similar profile within this sub-group, while the Ch. reticulatus venom showed a specific profile, displaying differences between families. Compounds from Buthidae venoms elute below 38% of ACN, while the non-Buthidae compounds elutes up to 50 % of ACN or 60% of ACN for O. elatus and Ch. reticulatus respectively. In all cases, we selected well defined peaks for the MS/MS analysis (arrows in Figure 2).

### MS/MS analysis

**Figure 3.**
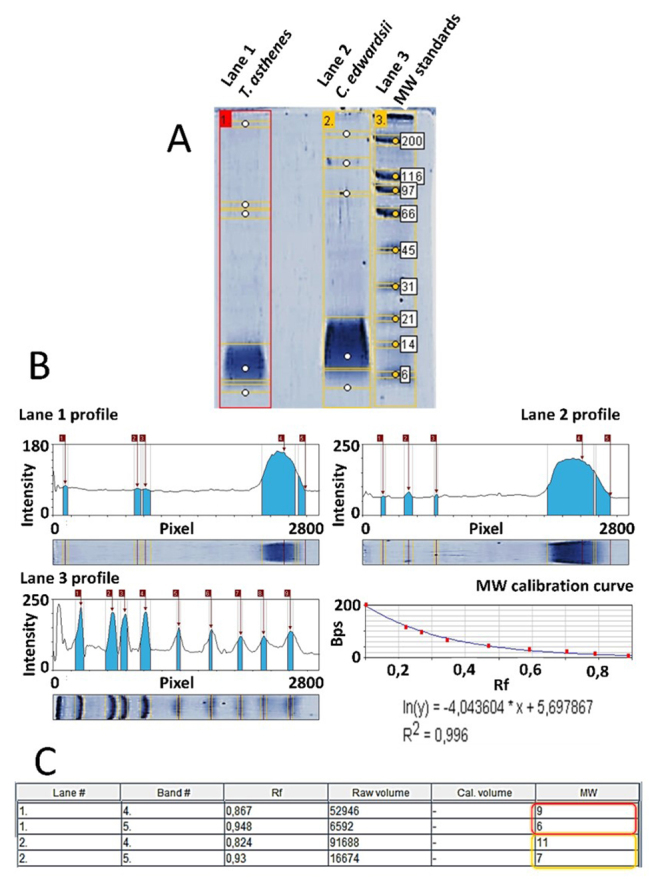
(A) TRIS-TRICINE electrophoresis analysis of *T. asthenes* (lane #1) and *C. edwardsii* (lane #2) venoms. (B) Intensity profile of each detected lane and bands in the TRIS-TRICINE electrophoresis gel. The bottom of each image shows the respective electrophoretic run. The intensity of each band is reported in arbitrary units. (C) Estimated molecular weights of bands detected below 6.5 kDa, red box for lane #1 (*T. asthenes*) and yellow box for lane #2 (*C. edwardsii*). Molecular weight estimation according the MW calibration curve analyzed with a simple exponential fit approximation with a R^2^ of 0.99. Rf: retention factor, MW: estimated molecular weight.

From all selected peaks, we obtained internal peptide sequences, matching different proteins from Buthidae scorpions. Only in the venoms from C. edwardsii and T. asthenes we found internal peptides matching neurotoxins affecting ion channels. A TRIS-TRICINE gel corroborate the presence of compounds with an estimated molecular masses similar to these neurotoxins (around to 6.5 kDa) in both venoms (see Figure 3). 

**Figure 4.**
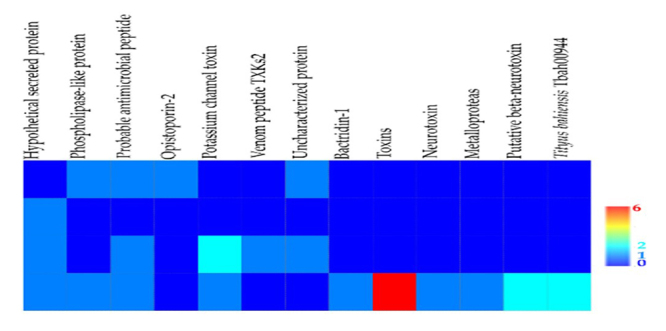
Matrix plot performed with presence/absence data of matched peptide family with MS/MS peptide sequence found in *Chactas reticulatus, Opisthacanthus elatus*, *Centruroides edwardsii* and *Tityus asthenes* venoms.

The MS/MS analysis of all scorpion venoms showed toxins similar to neurotoxins affecting potassium or sodium channels, beta-neurotoxin, antimicrobial peptides and metalloproteinase-like or phospholipase-like fragments (Figure 4). 

In Ch. reticulatus venom we found only one sequence that matched a hypothetical secreted protein from Hottentotta judaicus (Table 1). From the venom of O. elatus, we detected different sequences matching antimicrobial peptides, scorpine-like peptides and opistoporin, additional to the previous reported sequence matching a phospholipase A2 from O. cayaporum (also found here) (Table 1). These compounds were all previously reported in the venom of O. cayaporum. Of the six fragments detected in C. edwardsii, three sequences showed similarity with three different peptides affecting ion channels. One fragment matched a potassium channel toxin alpha-KTx 2.2 from Centruroides margaritatus and other with a potassium channel toxin alpha-KTx 2.1 from Centruroides noxius (Table 1). Other sequences showed similarity with peptides from Hottentotta judaicus and Mesobuthus gibbosus. Venom from T. asthenes showed more than 26 hits with different proteins and peptides and the main matched organism of T. asthenes sequences belongs to a species from the same genus, Tityus discrepans (Table 1). As observed in the electrophoresis, with compounds in the range of 14-31 kDa and 31-45 kDa, some fragments from T. asthenes matched metalloproteinases (venom metalloprotease-1) and phospholipases from other Buthidae (Mesobuthus eupeus) and Hormuridae (Opisthacanthus cayaporum) scorpions.

**Table 1.** *Chactas reticulatus, Opisthacanthus elatus*, *Centruroides edwardsii* and *Tityus asthenes* MS/MS analysis and the respective matching similar peptides or proteins. All MS/MS derived sequences correspond to internal peptides. All database numbers are from UniProtKB.

Fragment given name	MS/MS peptide sequence	Score/Identity	Matching peptide acc. number	Matched peptide family or name	Expected peptide m/z	Z	Calculated peptide mass	Matched organism
*Chactas reticulatus*								
ChrP1a	*K-ISSV[IN]NKDK-I	26-91% [I]/ 26-100% [N]	F1CJ08	Hypothetical secreted protein	523.775	+2	1045.54 £	*Hottentotta judaicus*
*Opisthacanthus elatus*								
Oe1	K-YGITNDSFFTK-L	226/100%	C5J8D1	Phospholipase-like protein (fragment)	646,806	+2	1291,597	*Opisthacanthus cayaporum*
Oe2	K-KAWNSPLANELK-S	156/100%	C7C1L2	Probable antimicrobial peptide	457,584	+3	1369,731	*Opisthacanthus cayaporum*
Oe3	- GWINEEKIQK-K	117/100%	P86121	Scorpine-like (fragment)	622,835	+2	1243,655	*Opisthacanthus cayaporum*
Oe4	R-KLGAQAMTDFIK-L	28/100%	C5J889	Putative uncharacterized protein (fragment)	661,859	+2	1321,704	*Opisthacanthus cayaporum*
Oe5	K-NFVAEKIGATPS-	18/100%	P83314	Opistoporin-2	617,325	+2	1232,636	*Opisthacanthus cayaporum*
*Centruroides edwardsii*								
CedP1a	K-AQFGQSAGAK-C	717/100%	P40755	Potassium channel toxin alpha-KTx 2.2	482.727	+2	963.477	*Centruroides margaritatus*
CedP2a	*TIINVKCTSPK-Q	13/100%	P08815	Potassium channel toxin alpha-KTx 2.1	645.371	+2	1288.706	*Centruroides noxius*
CedP3a	*K-ISSV[IN]NKDK-I	26-91% [I] / 26-100% [N]	F1CJ08	Hypothetical secreted protein	523.775	+2	1045.54 £	*Hottentotta judaicus*
CedP4a	*R-SGTPEKER-E	15/100%	P0DMI9	Antimicrobial peptide HsAp3	473.236	+2	944.456	*Heterometrus spinifer*
CedP5a	K-TKNETGFCLGPNGNEK-C	15/100%	F1CJ17	Venom peptide TXKs2	598.949	+3	1793.825	*Hottentotta judaicus*
CedP6a	*K-EETWCNLIYIKK-K	14/100%	A0A059UED3	Uncharacterized protein	417.720	+4	1666.828	*Mesobuthus gibbosus*
*Tityus asthenes*								
TaP1a	*KDGYIIEHR-G	1259/100%	P0CF39	Bactridin-1	586.082	+2	1171.599	*Tityus discrepans*
TaP1b	*K-KGSSGYCAWPACWCYGLPDNVK-I				860.393	+3	2578.133	
TaP2a	K-IFDYYNNK-C	350/100%	C9X4K1	Toxin TdNa3	538.775	+2	1075.497	*Tityus discrepans*
TaP3a	*R-KDGYLYDSK-N	53/100%	P0CI50	Neurotoxin LmNaTx15.1	565.796	+2	1129.529	*Lychas mucronatus*
TaP4a	ADDDLEGFSEEDLKAIK-E	335/100%	P0DL22	Toxin Tpa3	632.303	+3	1893.884	*Tityus pachyurus*
TaP5a	R-NKINGMK-F	142/100%	E4VNZ7	Venom metalloprotease-1	402.711	+2	803.432	*Mesobuthus eupeus*
TaP5b	R-ALDQDLELR-L				536.775	+2	1071.556	
TaP5c	*R-TGSKDCPASDGYIMGDR-N				937.885	+2	1873.782	
TaP6a	K- GTFCAEECTR-M	141/100%	P60215	Toxin To4	602.750	+2	1203.49	*Tityus obscurus*
TaP7a	K-SEYACPVIDK-F	93/100%	Q0GY43	Potassium channel toxin	584.777	+2	1167.548	*Tityus discrepans*
TaP8a	K-VWDYYNNK-C	81/100%	H1ZZI1	Toxin To12	551.245	+2	1100.493	*Tityus obscurus*
TaP9a	K-DGYLMEHDGCK-L	66/100%	P0DL23	Putative beta-neurotoxin	656.276	+2	1310.527	*Tityus pachyurus*
TaP9b	*NKDGYLMEHDGCK-L				532.556	+3	1594.676	
TaP10a	K-LEPAGDILAK-D	58	A0A0C9QKU3	*Tityus bahiensis* Tbah00944	513.782	+2	1025.576	*Tityus bahiensis*
TaP11a	K-YGITNDSFFTK-L	54/100%	C5J8D1	Phospholipase-like protein	646.810	+2	1291.609	*Opisthacanthus cayaporum*
TaP12a	*K-ISSV[IN]NKDK-I	26-91% [I]/ 26-100% [N]	F1CJ08	Hypothetical secreted protein	523.767	+2	1045.54 £	*Hottentotta judaicus*
TaP13a	K-VWDRATNK-C	32/100%	P15226	Beta-mammal/insect toxin Ts1/Voltage-gated sodium channels (Nav) gating-modifier.	495.267	+2	988.509	*Tityus serrulatus*
TaP13b	*K-KGSSGYCAWPACYCYGLPNWVK-V				876.402	+2	2626.169	
TaP14a	K-KAWNSPLANELK-S	31/100%	C7C1L2	Probable antimicrobial peptide	457.569	+3	1369.735	*Opisthacanthus cayaporum*
TaP15a	K-EGYPLNSSNGCK-S	24/100%	P0DL25	Putative beta-neurotoxin	656.800	+2	1311.577	*Tityus pachyurus*
TaP16a	R-DGYPVDEK-G	19/100%	P58296	Toxin Cn11	461.707	+2	921.408	*Centruroides noxius*
TaP16b	K-LSCLINDK-W				475.238	+2	948.495	
TaP16c	K-WCNSACHSR-G				576.246	+2	1150.465	
TaP16d	*ARDGYPVDEK-G				596.307	+2	1190.557	
TaP16e	K-YGYCYTGGLACYCEAVPDNVK-V				807.673	+3	2420.037	
TaP17a	*KDGYLVGTDGCK-Y	18/100%	P60262	Toxin TdII-3	671.327	+2	1340.628	*Tityus discrepans*

Z: indicates charge, while m/z indicates relation between mass and charge. £: indicates molecular weight calculated with asparagine residue and with lysine PTM. *Fragments with (K) acetylations, the molecular weight includes the PTM. Amino acids in square brackets indicate that we detected sequences containing both amino acids at that position.

**Figure 5.**
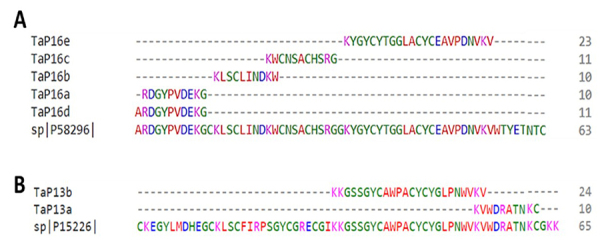
(A) Pairwise alignment of fragments TaP16a, TaP16b, TaP16c, TaP16d, TaP16e, from *T. asthenes*, matching peptide Cn11 (sp|P58296|) from *Centruroides noxius*, sodium channels blocker (Nav). (B) Pair-wise alignment of fragments TaP13a and TaP13b from *T. asthenes*, matching mature peptide beta-mammal/insect toxin Ts1 a voltage-gated sodium channels (Nav) gating-modifier.

The MS analysis of T. asthenes allowed the detection of sequences covering above 50% of two toxins affecting sodium channels. Six fragments matched one toxin affecting sodium channels (NaV) from Centruroides noxius (Toxin Cn11), covering 84% of this toxin (Figure 5A). Additionally, two more fragments, with a similarity of 100%, covered 50% of the toxin Ts1 from T. serrulatus (Buthidae), a proven voltage-gated sodium channel (Nav) gating-modifier (see Figure 5B). 

### Bioinformatics analysis

**Figure 6.**
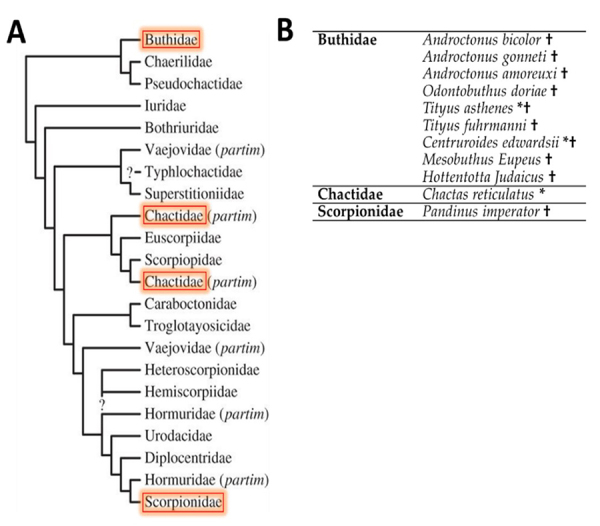
(A) Families and (B) species where the fragment KISSV[AIVSN]NKDKI had been reported. *Species published in this study; †: match with query peptide. Distribution of scorpion families according to Sharma et al. [35]. Figure adapted from Sharma et al. [35].

In all venoms, except O. elatus, we found a sequence fragment- KISSV[IN]NKDKI - with residue number six varying between I (isoleucine) or N (asparagine), depending on the species. The search for similar sequences that included the fragment, without specifying the residue in position number six [X], did match with a sequence of Hottentotta judaicus with accession number F1CJ08. When the search was performed with the residue "A", it matched with seven sequences, five of Androctonus bicolor (A0A0K0LCB5, A0A0K0LCC6, A0A0K0LCC9, A0A0K0LCD6 and A0A0K0LCD7), one of Odontobuthus doriae (A0A0U3YCW0), and one of Mesobuthus eupeus (E4VP36). Finally, with one of the residues “IVSN”, it matched with a previously reported fragments in the venoms of Androctonus amoreuxi, Pandinus imperator, Tityus fuhrmanni and Grosphus grandidieri [34]. Figure 6 shows the families and species where the fragment has been reported before.

### Evaluation of the physicochemical properties

Predicted physicochemical properties of - KISSV[AIVSN]NKDKI - based in the amino acid content and the net charge of this peptide, indicates that the fragment may be part of an antimicrobial protein (Table 2). Despite all possible amino acids sequences detected in the KISSV[X]NKDKI peptide, all conformations show a net predicted charge of 2+ and a similar hydrophobic residue percentage. Only peptides with residues KISSV[AIV]NKDKI are predicted to form an alpha helix showing a higher hydrophobic residue percentage. According to our MS/MS results, only KISSV[X]NKDKI with the Isoleucine (I) amino acid residue in the sequences from Chactas reticulatus, Centruroides edwardsii and Tityus asthenes may enhance antimicrobial activity in these venoms.

**Table 2.** Physicochemical properties of the KISSV[AIVSN]NKDKI peptide calculated in APD3 (Antimicrobial Peptide Calculator and Predictor).

Physicochemical properties	MS/MS peptide sequence				
	A**	I**	V**	S*	N*
Length (residues)	11	11	11	11	11
Molecular weight (MW)	1.202.406	1.244.487	1230.46	1.218.405	1.218.405
Net charge at physiological PH (7.4)	2	2	2	2	2
Hydrophobic residues (%)	36	36	36	27	27
Borman Index (kcal/mol)	2.1	1.81	1.89	2.57	2.57
Similar Peptide /%	AP01814/45.45	AP02863/ 42.85	AP02863/ 41.66		

*According to APD3 prediction, this peptide cannot form an alpha helix that is long enough to be an AP. **Predicted short alpha-helical cationic antimicrobial peptide.

## Discussion

Peptides are the dominant components of scorpion venoms and the primary source of their pharmacological diversity, becoming a natural source of bioactive compounds [6,36]. For this reason, scorpions are the focus of different studies attempting to describe the peptide content of their venoms in recent years. Although most scorpion venom electrophoresis are carried out using TRIS-TRICINE gels to visualize low molecular mass compounds, using SDS-PAGE gels allowed us to see the rich content of HMMC in each species.

Buthidae venoms from Colombia are rich in peptides affecting ionic channels and peptides with antimicrobial activity [34]. Although *Tityus* and *Centruroides* are widely distributed and studied in Colombia, most of the studies are focused on epidemiological aspects. Only four studies are available analyzing the composition of the venom of these two epidemiologically relevant scorpion genera; two characterizing the venom from *T. pachyurus*, one characterizing the peptide content of *Tityus macrochirus*, and one reporting an intraspecific difference in the biochemical and biological activity of *C. edwardsii* venom from two populations in different regions in Colombia [16]. In the former, the authors described the presence of a potent potassium-channel blocker and putative sodium scorpion channel toxins (NaScTxs) in the *Tityus* genus [18,20]. The present work indicates that *T. asthenes* and *C. edwardsii* seems to be an important source of toxins affecting ionic channels. Matched toxins from *T. asthenes* and *C. edwardsii* correspond to neurotoxins reported in other Buthidae scorpions like *Tityus discrepans*, *Tityus pachyurus*, *Tityus obscurus*, *Centruroides margaritatus* or *Centruroides noxius*, from Colombia, México, Venezuela or Brazil [18,37]. In *T. asthenes* we detected 16 different fragments from this venom (TaP2a, TaP3a, TaP6a, TaP7a, TaP8a, TaP9a, TaP9b, TaP13a, TaP13b, TaP15a, TaP16a, TaP16b, TaP16c, TaP16d, TaP16e, TaP17a), matching nine different toxins affecting sodium channels and one affecting potassium channels [37-39]. All these toxins, reported in other scorpions, can inhibit sodium currents, inhibit the inactivation of the activated channels, affect sodium channel activation by shifting the voltage of activation toward more negative potentials, or block the voltage-gated potassium channels. Some of these toxins were described in other *Tityus* species from different countries. Fragments TaP16a, TaP16b, TaP16c, TaP16d, TaP16e, matching 84% of the Cn11 amino acid sequence from *C. noxius* (Buthidae) and fragments TaP13a and TaP13b covering 50% of the toxin Ts1 from *T. serrulatus* (Buthidae) with a similarity of 100% in both cases, indicating the presence of sodium channel blockers in this venom. In the venom of *C. edwardsii,* two more fragments (CedP1a and CedP2a) were detected, matching peptides affecting potassium channels, and were described as potent inhibitors of voltage-gated potassium channels [40,41]. Although there is no previous report of the peptide or protein content of these two Buthidae scorpions from Colombia (*T. asthenes* and *C. edwardsii*), venom characterization of other Buthidae indicates that neurotoxins are the major content of these venoms, just as seen in our results [42-44]. We found four more fragments (TaP1a, TaP1b, TaP14a and CedP4a) from *T. asthenes* and *C. edwardsii* that are similar to antimicrobial peptides (AMP), active against Gram-negative and Gram-positive bacteria and fungi [13]. Many AMP have been described before in scorpion venoms [3,45], but this is the first report of the presence of these bio-active compounds in scorpion venoms from Colombia. Venom content from *O. elatus* seems to be very similar to that reported in *O. cayaporum* from Brazil [46]. All five fragments matched toxins reported in *O. cayaporum*, including a phospholipase-like protein and a probable antimicrobial peptide [46]. The *O. elatus* phospholipase region analyzed seems to be not exclusive for the elution of this proteins but also antimicrobial peptides. We expected to find many more sequences in major fraction in the *Ch. reticulatus* venom (as observed in the *T. asthenes* venom), the lack of recovered peptide families may be due to their absence, or due to there being very few *Chactas* sequences in the reference database.

SDS-PAGE and TRIS-TRICINE electrophoresis together with the MS/MS analysis allowed the detection of different HMMC and low molecular mass compounds (LMMC) in both *Tityus* and *Centruroides* venoms matching molecular weighs similar to neurotoxins, phospholipases or metalloproteinases. In Colombia, this HMMC had only been described in the venom from the scorpion *Opisthacanthus elatus* and the spider *Pamphobeteus verdolaga* [17,47], but never in Buthidae scorpions. HMMC are quite commonly distributed proteins in arachnids. Their main biological activities include housekeeping functions or enzymatic activities, like phospholipases or hyaluronidase [48-52]. Despite the clinical importance of these proteins, they are among the less studied venom components. These HMMC had been previously reported in the venoms from *T. bahiensis*, where the 32.69% of its venom content correspond to metalloproteinases [42]. 

In all cases, further proteomic studies are necessary to complete the MS/MS analysis of these important sources of bioactive compounds. 

In three of the four venoms analyzed by mass spectrometry, we detected a common fragment with a variant in the 6^th^ amino acid residue where a Isolecucine (I) can be replaced by an Asparagine (N) (K-ISSV[IN]NKDK-I) with a PTM with (K) acetylations. This fragment matches a hypothetical secreted protein from *Hottentotta judaicus* with unknown biological activity. We previously detected the same fragment in the venoms from *Pandinus imperator*, *Grosphus grandidieri*, *Tityus fuhrmanii* and *Androctonus amoreuxi* with an additional variant in the 6^th^ amino acid residue where the amino acids mentioned above can be replaced by Alanine (A), Valine (V) or cysteine (C) [34]. Is very important to consider that according to prediction results, only KISSV[X]NKDKI with the Isoleucine (I) amino acid residue in their sequences from *Chactas reticulatus, Centruroides edwardsii* and *Tityus asthenes* may enhance an antimicrobial activity in these venoms.

Currently, 20 scorpion families are recognized [35,53], and only 45 species have a transcriptomic analysis available, including Buthidae (with 22 species) and non-buthidae families (with 23 species). Considering that the Buthidae family has the highest number of species with a transcriptome available, proteins similar with our peptide (KISSV[IN]NKDKI) have mainly been described in the family Buthidae, with some reports in the Chactidae and Scorpionidae families. The presence of this protein mainly in the Buthidae family may suggests a recruitment of this peptide before Buthidae split from non-buthid species, as suggested by He et al. [54] for *Chaerilus tricostatus and Chaerilus tryznaithe* (Chaerilidae). Their evolutionary analysis showed that the NaTx, β-KTx, and bpp-like toxin types were recruited into the venom before the lineage split between Buthidae and non-Buthidae families. Similarly, Ma et al. [55] studied the evolution of the scorpion venom by comparative transcriptome analysis of venom glands and phylogenetic analysis of shared types of venom peptides and proteins between buthids and euscorpiids. This analysis revealed that at least five of the seven common types of venom peptides and proteins were likely recruited into the scorpion venom proteome before the lineage split between Buthidae and Euscorpiidae (i.e. basal in extant scorpions) with their corresponding genes undergoing individual or multiple gene duplication events. 

## Conclusion

The analyzed Buthidae venoms from Colombia may be considered a rich source of peptides similar to toxins affecting ionic channels. The *Opisthacanthus elatus* phospholipase region is composed not only of phospholipases but also of peptides with compounds similar to antimicrobial peptides. An interesting predicted antimicrobial peptide was detected in three of the analyzed venoms. When compared with the literature, this peptide is present in other scorpion families indicating a probable ancient peptide. The search for similar proteins that match with query peptides suggest that multiple types of venom peptides, including antimicrobial peptides, could have been recruited into the venom proteome during, before or at the basal split in the phylogeny of extant scorpions. In all cases, further proteomic studies are necessary to complete the MS/MS analysis of these important sources of bioactive compounds.

## References

[B1] van der Meijden A, Koch B, van der Valk T, Vargas-Muñoz LJ, Estrada-Gómez S (2017). Target-Specificity in Scorpions; Comparing Lethality of Scorpion Venoms across Arthropods and Vertebrates. Toxins (Basel).

[B2] Ortiz E, Gurrola GB, Schwartz EF, Possani LD (2015). Scorpion venom components as potential candidates for drug development. Toxicon.

[B3] Vargas LJ, Estrada-Gomez S (2014). Purification and Characterization of Venom Components as Source for Antibiotics. Mini-Rev Org Chem.

[B4] Vargas Munoz LJ, Estrada-Gomez S, Escobar J (2015). Snake and scorpion toxins venoms, a natural source of molecules with antimicrobial activity. CURARE.

[B5] Almaaytah A, Tarazi S, Mhaidat N, Al-Balas Q, Mukattash TL (2013). Mauriporin, a Novel Cationic α-Helical Peptide with Selective Cytotoxic Activity Against Prostate Cancer Cell Lines from the Venom of the Scorpion Androctonus mauritanicus. Int J Pept Res Ther.

[B6] Estrada G, Villegas E, Corzo G (2007). Spider venoms: a rich source of acylpolyamines and peptides as new leads for CNS drugs. Nat Prod Rep.

[B7] Rash LD, Hodgson WC (2002). Pharmacology and biochemistry of spider venoms. Toxicon.

[B8] Cao LY, Dai C, Li ZJ, Fan Z, Song Y, Wu YL (2012). Antibacterial Activity and Mechanism of a Scorpion Venom Peptide Derivative in vitro and in vivo. Plos One.

[B9] Cao LY, Li ZJ, Zhang RH, Wu YL, Li WX, Cao ZJ (2012). StCT2, a new antibacterial peptide characterized from the venom of the scorpion Scorpiops tibetanus. Peptides.

[B10] Cociancich S, Goyffon M, Bontems F, Bulet P, Bouet F, Menez A (1993). Purification and characterization of a scorpion defensin, a 4kDa antibacterial peptide presenting structural similarities with insect defensins and scorpion toxins. Biochem Biophys Res Commun.

[B11] Corzo G, Escoubas P, Villegas E, Barnham KJ, He WL, Norton RS (2001). Characterization of unique amphipathic antimicrobial peptides from venom of the scorpion Pandinus imperator. Biochem J.

[B12] Corzo G, Villegas E, Gomez-Lagunas F, Possani LD, Belokoneva OS, Nakajima T (2002). Oxyopinins, large amphipathic peptides isolated from the venom of the wolf spider Oxyopes kitabensis with cytolytic properties and positive insecticidal cooperativity with spider neurotoxins. J Biol Chem.

[B13] Diaz P, D'Suze G, Salazar V, Sevcik C, Shannon JD, Sherman NE (2009). Antibacterial activity of six novel peptides from Tityus discrepans scorpion venom. A fluorescent probe study of microbial membrane Na+ permeability changes. Toxicon.

[B14] Miyashita M, Sakai A, Matsushita N, Hanai Y, Nakagawa Y, Miyagawa HA (2010). Novel Amphipathic Linear Peptide with Both Insect Toxicity and Antimicrobial Activity from the Venom of the Scorpion Isometrus maculatus. Biosci Biotechnol Biochem.

[B15] Ramirez-Carreto S, Quintero-Hernandez V, Jimenez-Vargas JM, Corzo G, Possani LD, Becerril B (2012). Gene cloning and functional characterization of four novel antimicrobial-like peptides from scorpions of the family Vaejovidae. Peptides.

[B16] Estrada-Gomez S, Cupitra NI, Arango WM, Munoz LJ (2014). Intraspecific variation of centruroides edwardsii venom from two regions of Colombia. Toxins (Basel).

[B17] Estrada-Gómez S, Vargas Muñoz LJ, Saldarriaga-Córdoba M, Quintana Castillo JC (2016). Venom from Opisthacanthus elatus scorpion of Colombia, could be more hemolytic and less neurotoxic than thought. Acta Trop.

[B18] Barona J, Batista CV, Zamudio FZ, Gomez-Lagunas F, Wanke E, Otero R (2006). Proteomic analysis of the venom and characterization of toxins specific for Na+ - and K+ -channels from the Colombian scorpion Tityus pachyurus. Biochim Biophys Acta.

[B19] Rincón-Cortés CA, Olamendi-Portugal T, Carcamo-Noriega EN, Santillán EG, Zuñiga FZ, Reyes-Montaño EA (2019). Structural and functional characterization of toxic peptides purified from the venom of the Colombian scorpion Tityus macrochirus. Toxicon.

[B20] Guerrero-Vargas JA, Mourão CBF, Quintero-Hernández V, Possani LD, Schwartz EF (2012). Identification and Phylogenetic Analysis of Tityus pachyurus and Tityus obscurus Novel Putative Na(+)-Channel Scorpion Toxins. PloS One.

[B21] Otero R, Navio E, Cespedes FA, Nunez MJ, Lozano L, Moscoso ER (2004). Scorpion envenoming in two regions of Colombia: clinical, epidemiological and therapeutic aspects. Trans R Soc Trop Med Hyg.

[B22] Ward MJ, Ellsworth SA, Nystrom GS (2018). A global accounting of medically significant scorpions: Epidemiology, major toxins, and comparative resources in harmless counterparts. Toxicon.

[B23] Gómez JP, Quintana JC, Arbeláez P, Fernández J, Silva JF, Barona J (2010). Picaduras por escorpión Tityus asthenes en Mutatá, Colombia: aspectos epidemiológicos, clínicos y toxinológicos. Biomedica.

[B24] Laemmli UK (1970). Cleavage of structural proteins during the assembly of the head of bacteriophage T4. Nature.

[B25] Doumas BT, Bayse DD, Borner DD, Carter RJ, Elevitch F, Garber CC (1981). A candidate Reference Method for determination of total protein in serum. II. Test for transferability. Clin Chem.

[B26] Doumas BT, Bayse DD, Carter RJ, Peters T, Schaffer R (1981). A candidate Reference Method for determination of total protein in serum. I. Development and validation. Clin Chem.

[B27] Zheng K, Wu L, He Z, Yang B, Yang Y (2017). Measurement of the total protein in serum by biuret method with uncertainty evaluation. Measurement.

[B28] Istvan LJ, Istvan LS (2021). GelAnalyzer 19.1.

[B29] Fernandez J, Gutierrez JM, Angulo Y, Sanz L, Juarez P, Calvete JJ (2010). Isolation of an acidic phospholipase A2 from the venom of the snake Bothrops asper of Costa Rica: biochemical and toxicological characterization. Biochimie.

[B30] Chen C, Li Z, Huang H, Suzek BE, Wu CH, UniProt Consortiium (2013). A fast Peptide Match service for UniProt Knowledgebase. Bioinformatics.

[B31] Wu CH, Yeh LSL, Huang H, Arminski L, Castro-Alvear J, Chen Y (2003). The Protein Information Resource. Nucleic Acids Res.

[B32] Chen C, Li Z, Huang H, Suzek BE, Wu CH, Consortium U (2013). A fast Peptide Match service for UniProt Knowledgebase. Bioinformatics.

[B33] Wang G, Li X, Wang Z (2016). APD3: the antimicrobial peptide database as a tool for research and education. Nucleic Acids Res.

[B34] Estrada-Gomez S, Gomez-Rave L, Vargas-Munoz LJ, van der Meijden A (2017). Characterizing the biological and biochemical profile of six different scorpion venoms from the Buthidae and Scorpionidae family. Toxicon.

[B35] Sharma PP, Fernández R, Esposito LA, González-Santillán E, Monod L (2015). Phylogenomic resolution of scorpions reveals multilevel discordance with morphological phylogenetic signal. Proc Biol Sci.

[B36] Saez NJ, Senff S, Jensen JE, Er SY, Herzig V, Rash LD (2010). Spider-venom peptides as therapeutics. Toxins (Basel).

[B37] D'Suze G, Schwartz EF, Garcia-Gomez BI, Sevcik C, Possani LD (2009). Molecular cloning and nucleotide sequence analysis of genes from a cDNA library of the scorpion Tityus discrepans. Biochimie.

[B38] Batista CV, del Pozo L, Zamudio FZ, Contreras S, Becerril B, Wanke E (2004). Proteomics of the venom from the Amazonian scorpion Tityus cambridgei and the role of prolines on mass spectrometry analysis of toxins. J Chromatogr B Analyt Technol Biomed Life Sci.

[B39] Batista CV, D'Suze G, Gomez-Lagunas F, Zamudio FZ, Encarnacion S, Sevcik C (2006). Proteomic analysis of Tityus discrepans scorpion venom and amino acid sequence of novel toxins. Proteomics.

[B40] Garcia-Calvo M, Leonard RJ, Novick J, Stevens SP, Schmalhofer W, Kaczorowski GJ (1993). Purification, characterization, and biosynthesis of margatoxin, a component of Centruroides margaritatus venom that selectively inhibits voltage-dependent potassium channels. J Biol Chem.

[B41] Grissmer S, Nguyen AN, Aiyar J, Hanson DC, Mather RJ, Gutman GA (1994). Pharmacological characterization of five cloned voltage-gated K+ channels, types Kv1.1, 1.2, 1.3, 1.5, and 3.1, stably expressed in mammalian cell lines. Mol Pharmacol.

[B42] de Oliveira UC, Candido DM, Dorce VA, Junqueira-de-Azevedo Ide L (2015). The transcriptome recipe for the venom cocktail of Tityus bahiensis scorpion. Toxicon.

[B43] de Paula Santos-da-Silva A, Candido DM, Nencioni ALA, Kimura LF, Prezotto-Neto JP, Barbaro KC (2017). Some pharmacological effects of Tityus obscurus venom in rats and mice. Toxicon.

[B44] Martin-Eauclaire MF, Pimenta AMC, Bougis PE, De Lima ME (2016). Potassium channel blockers from the venom of the Brazilian scorpion Tityus serrulatus (Lutz and Mello, 1922). Toxicon.

[B45] Vargas Munoz LJ, Estrada-Gomez S, Vasquez J (2015). Toxinas de venenos de serpientes y escorpiones, una fuente natural de moleculas con actividad antimicrobiana. CURARE.

[B46] Schwartz EF, Camargos TS, Zamudio FZ, Silva LP, Bloch C, Caixeta F (2008). Mass spectrometry analysis, amino acid sequence and biological activity of venom components from the Brazilian scorpion Opisthacanthus cayaporum. Toxicon.

[B47] Estrada-Gomez S., Vargas-Munoz L.J., Saldarriaga-Cordoba M., Cifuentes Y., Perafan C (2017). Identifying different transcribed proteins in the newly described Theraphosidae Pamphobeteus verdolaga. Toxicon.

[B48] Cheng TC, Long RW, Wu YQ, Guo YB, Liu DL, Peng L (2016). Identification and characterization of toxins in the venom gland of the Chinese bird spider, Haplopelma hainanum, by transcriptomic analysis. Insect Sci.

[B49] Jiang L, Peng L, Chen J, Zhang Y, Xiong X, Liang S (2008). Molecular diversification based on analysis of expressed sequence tags from the venom glands of the Chinese bird spider Ornithoctonus huwena. Toxicon.

[B50] Yuan C, Jin Q, Tang X, Hu W, Cao R, Yang S (2007). Proteomic and peptidomic characterization of the venom from the Chinese bird spider, Ornithoctonus huwena Wang. J Proteome Res.

[B51] Borges MH, Figueiredo SG, Leprevost FV, De Lima ME, Cordeiro MN, Diniz MR (2016). Venomous extract protein profile of Brazilian tarantula Grammostola iheringi: searching for potential biotechnological applications. J Proteomics.

[B52] Liao Z, Cao J, Li S, Yan X, Hu W, He Q (2007). Proteomic and peptidomic analysis of the venom from Chinese tarantula Chilobrachys jingzhao. Proteomics.

[B53] Santibáñez-López CE, Cid-Uribe JI, Batista CVF, Ortiz E, Possani LD (2016). Venom Gland Transcriptomic and Proteomic Analyses of the Enigmatic Scorpion Superstitionia donensis (Scorpiones: Superstitioniidae), with Insights on the Evolution of Its Venom Components. Toxins (Basel).

[B54] He Y, Zhao R, Di Z, Li Z, Xu X, Hong W (2013). Molecular diversity of Chaerilidae venom peptides reveals the dynamic evolution of scorpion venom components from Buthidae to non-Buthidae. J Proteomics.

[B55] Ma Y, He Y, Zhao R, Wu Y, Li W, Cao Z (2012). Extreme diversity of scorpion venom peptides and proteins revealed by transcriptomic analysis: implication for proteome evolution of scorpion venom arsenal. J Proteomics.

